# Control of Acinetobacter baumannii outbreak in intensive care units in Tunisia, 2022

**DOI:** 10.1093/eurpub/ckac131.390

**Published:** 2022-10-25

**Authors:** E Mziou, H Ghali, S Bhiri, A Ben Cheikh, R Bannour, M Ghribi, S Trabelsi, S Khefacha, M Ben Rejeb, H Said Latiri

**Affiliations:** 1 Department of Prevention and Security of Care, Sahloul University Hospital, Sousse, Tunisia; 2 Faculty of Medicine of Sousse, University of Sousse, Sousse, Tunisia; 3 Family Medicine, Faculty of Medicine of Sousse, Sousse, Tunisia; 4 LR20SP06, Emerging Bacterial Resistance in Hospitals Veterinarians and the Environment and Security of Care, Tunisia

## Abstract

**Introduction:**

Acinetobacter baumannii is an emerging pathogen that is increasingly resistant to antibiotics and is mainly responsible for pneumopathy in fragile patients. This germ is frequently responsible for epidemics in hospitals. We aimed to describe the steps of the investigation of an outbreak of Acinetobacter baumannii affected our hospital, the measures implemented and the follow-up of the actions.

**Methods:**

Following alerts issued by the microbiology department concerning 5 swabs detecting Acinetobacter of the same strain and the same antibiotic resistance profile in 3 different departments of intensive-care units (ICU), a team of the prevention and healthcare security department went onsite for an investigation in the hospital.

**Results:**

We identified five cases with identical strains of multi-resistant Acinétobacter. The field visit allowed to identify some deficiencies in professional practices. All the patients were hospitalized in ICU (medical and surgical). The synoptic table showed that there was an overlap of hospitalization periods.A crisis cell was set up to validate, coordinate and implement control measures in accordance with CTINILS recommendations. Indeed, we proceeded to a technical isolation of the cases in their hospitalization sector, reinforced the basic hygiene and bio-cleaning measures and sensitized the medical and paramedical. Given that the three ICU departments shared the same medical staff during night shifts, the assumption that the germ was carried by the caregivers was the most likely hypothesis. We proceeded with a swab of the elements of the environment in the services concerned. Results showed that Acinetobacter was found on the nursing cart (visibly clean). A training about bio-cleaning and hygiene standard precautions is programmed.

**Conclusions:**

Continuous surveillance, continuous hygiene trainings, combined with a rapid reaction capacity in case of identification of a new case, is essential to control the spread of nosocomial germs.

**Key messages:**

• Multidrug-resistant Acinetobacter baumannii (MRAB) is an emerging cause of intensive care unit (ICU) outbreaks.

• Enhanced infection control measures limited the outbreak.

